# Advanced laser scanning for highly-efficient ablation and ultrafast surface structuring: experiment and model

**DOI:** 10.1038/s41598-018-35604-z

**Published:** 2018-11-26

**Authors:** Andrius Žemaitis, Mantas Gaidys, Marijus Brikas, Paulius Gečys, Gediminas Račiukaitis, Mindaugas Gedvilas

**Affiliations:** Center for Physical Sciences and Technology, Savanoriu Ave. 231, LT-02300 Vilnius, Lithuania

## Abstract

Ultra-short laser pulses are frequently used for material removal (ablation) in science, technology and medicine. However, the laser energy is often used inefficiently, thus, leading to low ablation rates. For the efficient ablation of a rectangular shaped cavity, the numerous process parameters such as scanning speed, distance between scanned lines, and spot size on the sample, have to be optimized. Therefore, finding the optimal set of process parameters is always a time-demanding and challenging task. Clear theoretical understanding of the influence of the process parameters on the material removal rate can improve the efficiency of laser energy utilization and enhance the ablation rate. In this work, a new model of rectangular cavity ablation is introduced. The model takes into account the decrease in ablation threshold, as well as saturation of the ablation depth with increasing number of pulses per spot. Scanning electron microscopy and the stylus profilometry were employed to characterize the ablated depth and evaluate the material removal rate. The numerical modelling showed a good agreement with the experimental results. High speed mimicking of bio-inspired functional surfaces by laser irradiation has been demonstrated.

## Introduction

Ultra-short laser pulses have shown their applicability for high-quality laser micromachining of metals^1–5^, semiconductors^6–8^ and insulators^9–11^ in scientific^3,12,13^, technological^14–21^, and medical^22–25^ applications. However, their real usage is limited by the low ablation rates at which material is removed. The experimental/theoretical work clarifying the optimization of ablation rate by the selection of a particular laser fluence emerged a decade ago^26^. Later, efficient laser ablation has been widely investigated by several scientific groups^27–36^. However, there are only a few attempts to analyse the ablation rate by taking into account the incubation effect and the saturation of the ablation depth for multi-pulse ablation^35,37^. The incubation phenomenon defines how the multi-pulse ablation threshold decreases with increasing number of pulses per spot^38,39^, and it was validated in many experimental occasions^40–42^. Nevertheless, despite the threshold fluence decrease with the increasing number of laser pulses, the depth of ablated dimple reaches a saturation value after a particular number of pulses applied^39,43–46^. The pioneering research works of efficient ablation suggested an optimal ratio of laser peak fluence to the ablation threshold close to *e*^2^ ≈ 7.4 for the most efficient ablation with maximal possible material removal rate^26,27^. The assumptions of efficient ablation model are: the laser beam has a Gaussian beam profile, laser ablation has threshold behaviour, and ablated depth is proportional to the logarithm of the fluence applied. The maximum volume of the paraboloid and most efficient ablation are achieved when the applied peak laser fluence is *e*^2^ times higher than the ablation threshold. This model is in good agreement with most of experimental works^27–35^. However, this model has a drawback when applied to trench ablation with a scanned laser beam. It suggests that an optimal beam scanning speed on the sample should be zero for the maximal material removal rate. This fact is in a contradiction with the experimental results, because ablation depth saturates for multi-pulse treatment^43–45^ and maximum trench depth is achieved for a non-zero scanning speed^47^. Therefore, the selection of the optimal beam scanning speed is still an open question. Also, the pioneering works of the efficient laser ablation were dedicated only to a parabolic dimple and trench formation^26,27^. However, there are no scientific works dedicated to the theoretical/experimental analysis of rectangular shaped cavity ablation and formation of multi-layer cavities with two-and-a-half-dimensional (2.5D) shape.

Here, we present the theoretical and experimental studies of the rectangular shaped cavity ablation taking into account the incubation phenomenon and the saturated ablation depth for multi-pulse treatment. The new model has been created for a scanned laser beam on the plane surface and ablation of the rectangular shape cavity. We applied ultra-short laser pulses to ablate the target material at various inter-pulse distances (beams scanning speeds) and laser fluences (spot sizes on sample). Proof-of-principle experiments on copper by various processing parameters sets demonstrate that an optimal point for highest ablation rate can be predicted by our theoretical model.

## Materials and Methods

### Experimental setup and procedures

**Figure 1:**
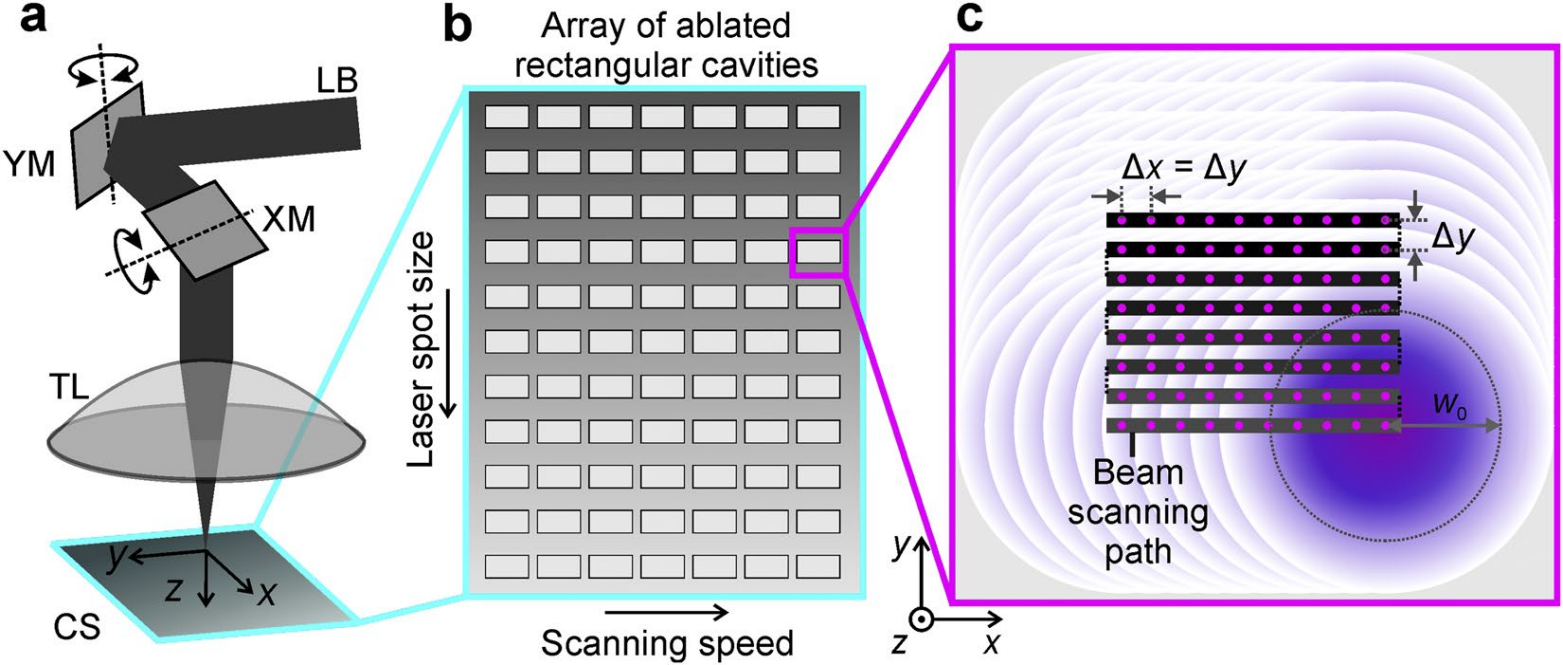
Experimental setup and procedures. (**a**) Principal scheme of the experimental setup: LB - laser beam; XM and YM - *x*- and *y*-mirrors controlled by galvanometric motors; TL - telecentric *f*-theta lens; CS - copper sample; *xyz* - coordinate system. (**b**) Schematic representation of an array of laser scanned rectangular areas on the copper sample with variable processing parameters: the beam scanning speed and the laser spot size on the sample. (**c**) Schematic representation of the laser beam scanning path in each of the squares: black step-type line represents the path of the scanned laser beam on the sample; solid dots on the beam’s scanning path represents the centre positions of Gaussian laser pulses; overlapping circles show overlapped laser spots; Δ*x* is the inter-pulse distance between laser spots equal to the ratio of the beam scanning speed and pulse repetition rate; Δ*y* is the distance between two adjacent scanned lines; *w*_0_ is the laser spot radius on the sample.

The industrial-grade diode-pumped solid-state laser (Atlantic, Ekspla) with a pulse duration of 10 ps emitting at a wavelength of 1064 nm was used in the experiments. The laser provided light pulses with pulse energy up to 130 μJ at a repetition rate of 100 kHz with an average laser power of 13.0 W. The Gaussian beam quality factors M^2^ declared by the laser manufacturer was of 1.062 and 1.043 in transverse directions *x* and *y*, respectively. The scheme of experimental setup is given in Fig. 1a.

The beam position on the sample was controlled by using a galvanometer scanner (Scangine 14, Scanlab). Translation of the laser spot on the target material at a controllable speed up to 300 mm/s provided the adjustable distance between the transverse irradiation spots Δ*x*. The telecentric *f*-theta objective lens with the focal length of 80 mm was used to focus the beam on the surface of the target material. The array of rectangular cavities with the transverse spatial dimensions of 2.5 mm × 1.0 mm and the depth-dependant on the processing parameters was ablated (Fig. 1b). The scanning speed of the beam was changed in a horizontal axis. The laser spot size on the sample was changed in the vertical axis of the array by controlling the elevation position *z* of the sample. The variable spot sizes provided different laser fluences. The path of the scanned beam on the copper sample consisted of parallel lines of overlapped laser pulses as shown in Fig. 1c. The distance between laser pulses was chosen to be identical with the distance between scanned lines as Δ*x* = Δ*y* in order to reduce the number of processing parameters. The ablation rate measurement experiments were conducted by using maximal available laser power of 13.0 W in order to have the maximal material removal rate.

### Laser beam characterization

**Figure 2:**
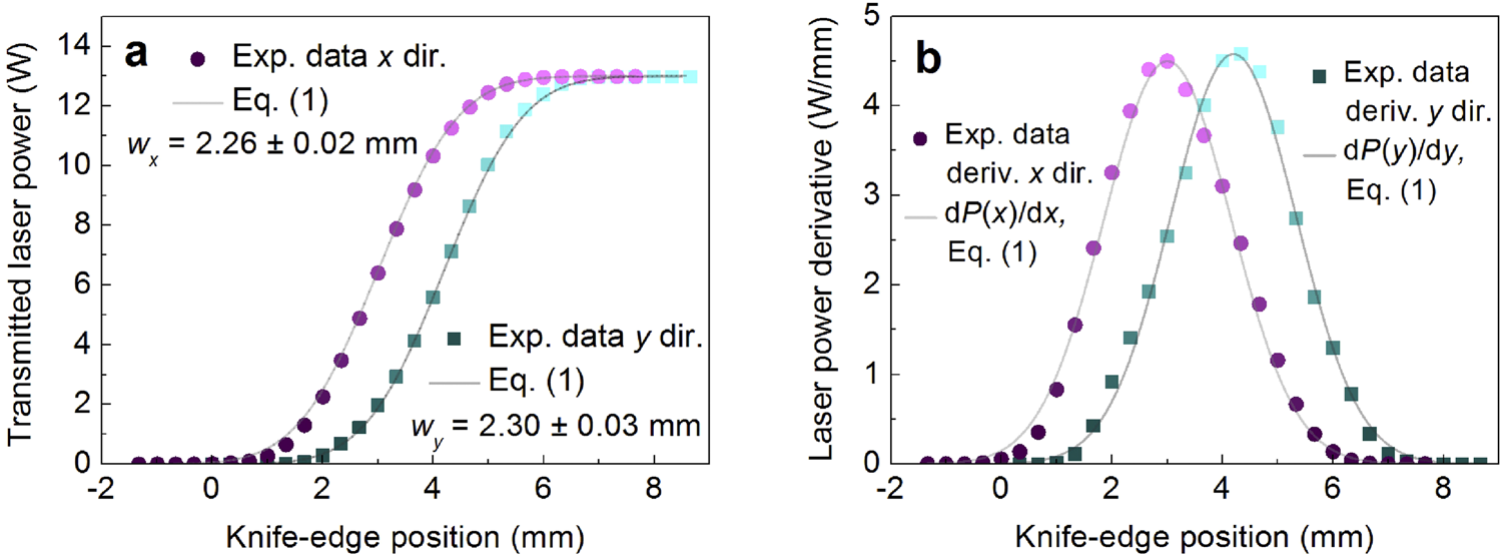
Beam profile characterization. (**a**) Transmitted laser power and (**b**) its first derivatives dependences on the knife-edge position in transverse *x* and *y* directions at the entrance of the galvanometer scanner. Circular and rectangular solid dots represent: (**a**) experimental data points; (**b**) its first derivative. Solid lines represent: (**a**) fits by Equation (1); (**b**) its first derivate.

The power meter (Nova ΙΙ, Ophir) equipped with a thermal power sensor (30A-BB-18, Ophir) was used to measure the average laser power. The profiles of the laser beam distribution measured by using the knife-edge method^48,49^ are given in Fig. 2.

The experimental data for the measured transmitted laser power *P*(*x*) was fitted using the equation^50^, which assumes a Gaussian intensity profile of the beam:

1 $$P(x)=\frac{{P}_{0}}{2}[1+{\rm{erf}}(\frac{\sqrt{2}(x-{x}_{{\rm{c}}})}{{w}_{x}})],$$

where *P*_0_ = 13.0 W is the laser power, *x*_c_ the centre coordinate of the Gaussian intensity profile, and erf is a standard error function. The result of experimental data points fit by Equation (1) are shown in Fig. 2a. The beam radiuses of *w*_*x*_ = 2.26 ± 0.02 mm and *w*_*y*_ = 2.30 ± 0.03 mm at 1/*e*^2^ level were received for *x* and *y* directions, respectively. The first derivatives of the measured transmitted laser power provide the Gaussian beam intensity distributions in the *x* and *y* directions as shown in Fig. 2b. It is clearly seen from fits of experimental data points by first derivative of Equation (1) in Fig. 2b that the beam has well defined Gaussian distribution in transverse plane. By performing goodness of fit testing (comparing how well experimental intensity profiles correlates with Gaussian function fits) the reduced chi-squared values of 0.012 and 0.011 were achieved for *x* and *y* directions, respectively. Those two values show that experimental data points deviate from Gaussian distribution fits only by 1.2% and 1.1%. Such Gaussian profiles of the beam confirms beam quality factor declared by laser manufacturer. A two-dimensional Gaussian beam at the entrance of the galvanometer scanner exhibits a Gaussian fluence distribution *F*(*x*,*y*) on the target material surface according to:

2 $$F(x,\,y)={F}_{0}{e}^{-\frac{2{(x-{x}_{0})}^{2}+2{(y-{y}_{0})}^{2}}{{w}_{0}^{2}}}$$

where *F*_0_ is the maximum fluence in the centre, *x*_0_ and *y*_0_ are the coordinates of the central point, *w*_0_ is the Gaussian beam radius. At radius *w*_0_, by definition *F*(*x*, *y*) decreases to *F*_0_/*e*^2^. The dependence of the peak fluence on the single-pulse energy can be written as

3 $${F}_{0}=\frac{2{E}_{{\rm{p}}}}{\pi {w}_{0}^{2}},$$

where *E*_p_ is the pulse energy.

**Figure 3:**
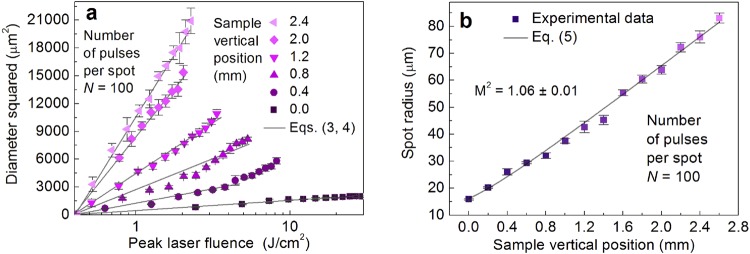
Gaussian beam spot size characterization. (**a**) Squared diameters *D*^2^ of the ablated areas of copper in dependence on the peak laser fluence *F*_0_ at a different sample vertical position *z* values. Solid dots - experimental data points, solid line - fit by Equations (3) and (4). (**b**) Gaussian beam spot radius dependence on sample vertical position *z* values. Solid dots - experimental data points, solid line - fit by Equation (5). Pulse duration *τ* = 10 ps, laser wavelength *λ* = 1064 nm, repetition rate *f*_p_ = 100 kHz.

The radius of the transverse focal spot on the target sample surface was measured by the technique described in^51^. Measuring technique provides information about the actual laser beam spot size despite the beam quality factor M^2^. It is valid for a Gaussian beam with a good beam qualify factor of M^2^ = 1.1^52^, moderate beam quality factor of M^2^ = 1.3^53–55^, and even for multimode beam with a poor beam quality factor of M^2^ = 95^56^. Also, this measuring technique is valid for a highly elliptical Gaussian beam^57^. Assuming that a laser beam has the Gaussian spatial beam profile, the relation between the crater diameter *D* and the peak laser fluence *F*_0_ in the centre of the Gaussian beam can be written as^51^:

4 $${D}^{2}=2{w}_{0}^{2}\,\mathrm{ln}(\frac{{F}_{0}}{{F}_{{\rm{th}}}(N)}),$$

where *w*_0_ is the radius of the Gaussian beam on the sample, *F*_th_(*N*) is the ablation threshold, *N* is the number of laser pulses applied to a single spot. The optical microscopy was employed for the characterization of ablated craters (dimples). Diameters and depths of craters ablated by the single and multi-pulse regimes in copper for determination of ablation threshold, incubation parameter and penetration depth have been performed by using an optical microscope (Eclipse LV100, Nikon) equipped with high-definition 5-megapixel CCD digital camera (DS-Fi1, Nikon) with a resolution of 2560 × 1920. The camera was equipped with a controller (Digital Sight DS-U2, Nikon) and image processing software (NIS-Elements D, Nikon). The objective (LU Plan Fluor 20x, Nikon) with a numerical aperture (NA) = 0.5 and a magnification factor of 20X was used in bright field. The specimen was illuminated by a halogen lamp (LV-HL50PC, Nikon). The pixel size of the digital microscope image was ≈0.2 µm, which was much smaller than the variation of crater size from different craters ablated under the same experimental conditions. Therefore, the standard deviation of five ablated craters was taken as a measurement error. By having Equation (4) with the pulse energy values, an ablation threshold pulse energy *E*_th_ is calculated from a semi-logarithmic plot of the diameter squared of the ablated area *D*^2^ versus pulse energy *E*_p_. The slope of the line yields the Gaussian beam radius *w*_0_. Taking into account the beam radius and Equation (3), the laser fluence values can be calculated. The results of ablation using picosecond laser treatment at different elevation position *z* of copper samples are presented in Fig. 3a.

The slope increased with increasing vertical position of the sample Fig. 3a. The laser spot radius was varied by changing the vertical position *z* of the sample from 0.0 mm to 2.6 mm by 0.2 mm. The Gaussian beam radiuses were calculated from the linear fit slopes by Equations (3) and (4). The spot radius dependence on the sample vertical position is given in Fig. 3b. The beam propagation equation along the *z*-direction for spot radius *w*(*z*) of non-ideal Gaussian beam can be written as^58^:

5 $$w(z)={w}_{0}{[1+{(\frac{(z-{z}_{0})\lambda {M}^{2}}{\pi {w}_{0}^{2}})}^{2}]}^{\frac{1}{2}},$$

where *w*_0_ is the spot size in focus, *λ* = 1064 nm is the wavelength of irradiation, *z*_0_ is the focal position, M^2^ is the beam quality parameter. From the experimental data fit by Equation (5) in Fig. 3b the Gaussian beam quality factor of M^2^ = 1.06 ± 0.01 was retrieved. The received beam quality parameter is in good agreement with the data provided by the laser manufacturer.

### Ablation rate characterization

**Figure 4:**
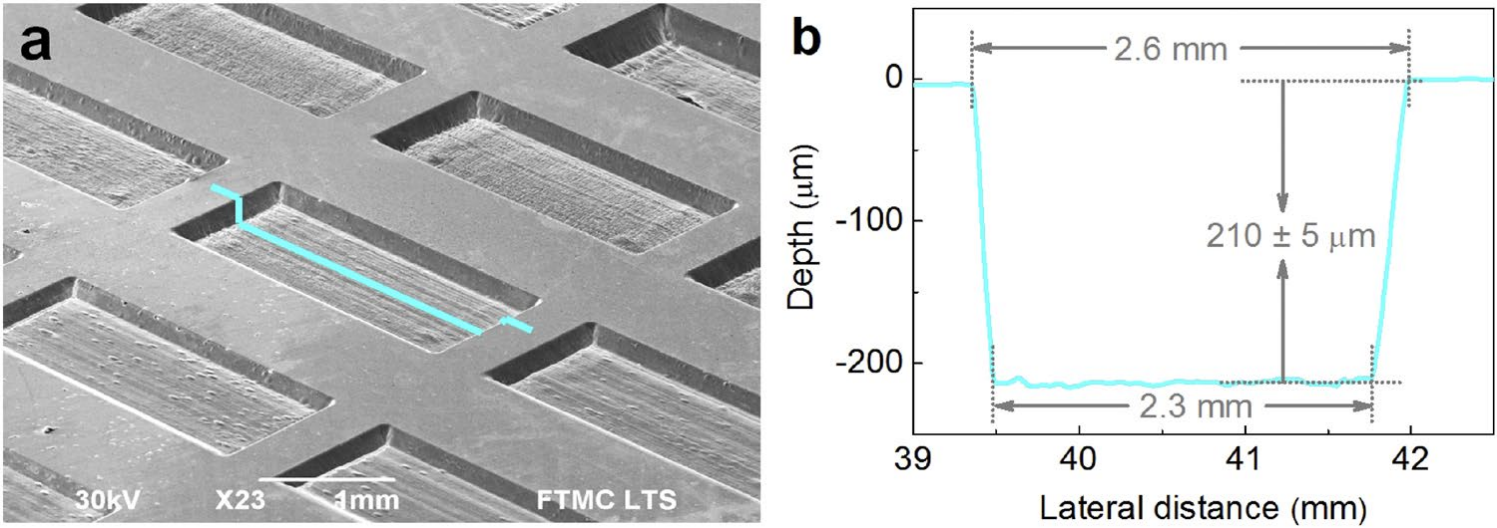
An array of laser ablation tests performed by a picosecond laser. (**a**) SEM image of an array of rectangular shaped cavities on copper ablated by using a picosecond laser. Lateral ablated cavity transverse dimensions of the ablated cavities are 2.5 mm × 1.0 mm; (**b**) measured profile of laser ablated cavity: average depth 210 ± 5 μm; width at the top 2.6 mm; width at the bottom 2.3 mm. Processing parameters: wavelength of irradiation *λ* = 1064 nm; mean laser power *P*_0_ = 13.0 W; repetition rate *f*_p_ = 100 kHz; pulse duration *τ* = 10 ps; inter-pulse distance *Δx* = 1.6 μm (*v* = 160 mm/s); peak laser fluence *F*_0_ = 3.05 J/cm^2^ (*w*_0_ = 52.1 μm), the number of scans 2.

The solid copper (CW004A, Ekstremalė) target plate with the dimensions of 50 mm × 50 mm × 5 mm, purity of 99.9%, and surface roughness of R_a_ < 0.1 µm (mirror finish) was used in the ablation tests. The surface topography was studied by using a scanning electron microscope (SEM) (JSM-6490LV, JEOL). The SEM micrograph of an array of laser ablated rectangular shaped cavities is shown in Fig. 4a.

The profiles of laser-ablated cavities were measured by a stylus profiler (Dektak 150, Veeco) with the measurement resolution of 0.1 μm, 1 μm and 1 nm in *x*, *y* and *z* directions, respectively. The typical profile is shown in Fig. 4b. The ablation rate was evaluated from the volume of the ablated cavities using data from the profiles. The measuring errors of ablation depth were related to the surface roughness of the cavity bottom. Therefore, the average relative error of ablation rate was less than 2.5%.

### Ablation model of rectangular shaped cavity

**Figure 5:**
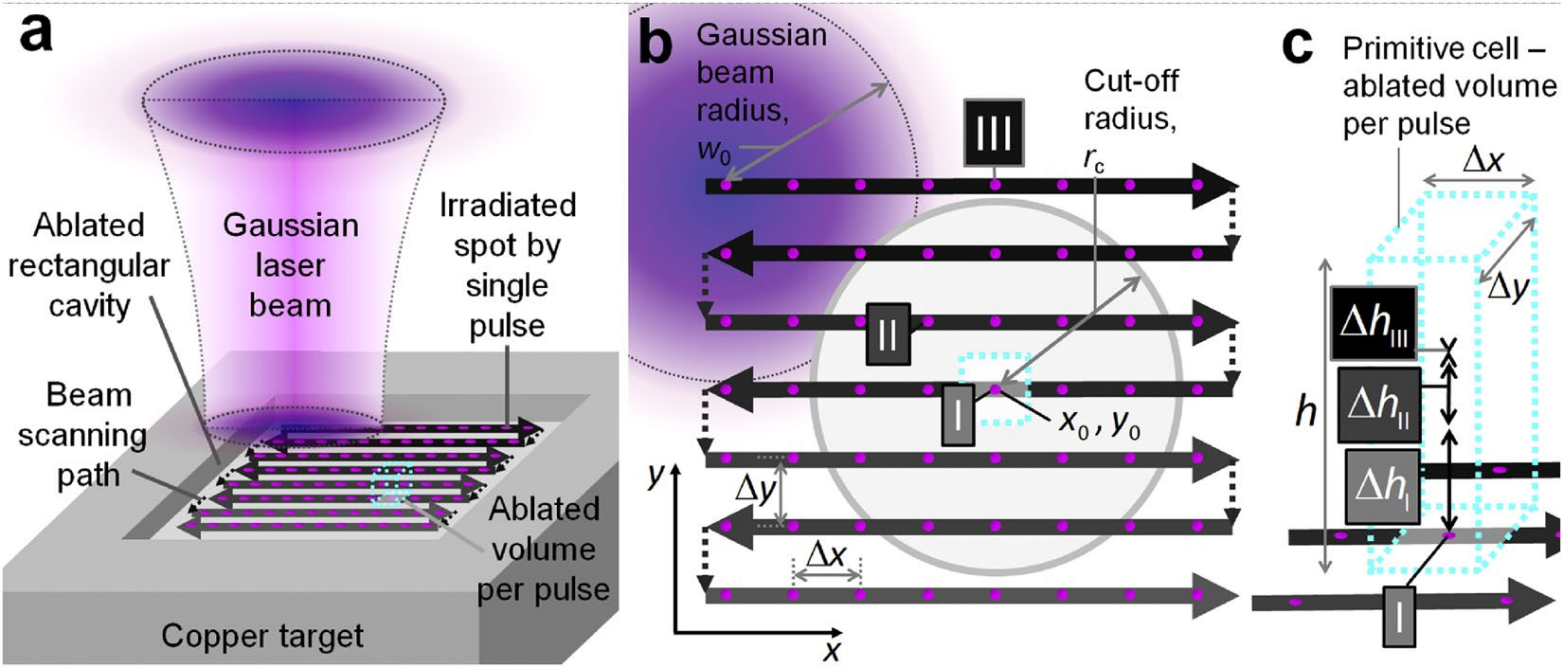
Graphical representation of the laser ablation model. (**a**) 3D scheme of the laser ablation model of a rectangular cavity by parallel lines of the scanned laser beam. Solid circles represent the array of spots irradiated by single laser pulses. The black line with arrows represents the beam scanning path and its direction on the sample. The cube in the centre by a dashed cyan line represents the primitive cell - volume ablated by a single pulse. However, the depth of the ablated primitive cell is influenced by many surrounding pulses. The primitive cell has a volume equal to the volume of the whole rectangular cavity divided by the total number of pulses applied. (**b**) View from the top. The beam is scanned from left to right and from top to bottom leaving a quadratic array of spots irradiated by single laser pulses. Δ*x* and Δ*y* represent the lateral distances between adjacent laser pulses and scanned lines, respectively. The single laser pulse ablates the area marked by the cyan dashed line rectangle with the lateral dimensions of Δ*x* · Δ*y* in the position (*x*_0_, *y*_0_) marked by (I). Grey circle with the centre coordinates (*x*_0_, *y*_0_) represents the active ablation area with the cut-off radius *r*_c_. The non-zero ablation depth is achieved from the pulses within an active circle (II). All the laser pulses in position (III) outside the cut-off area have zero ablation depth because the laser fluence is below the ablation threshold in position (I). (**c**) 3D representation of the ablated volume per pulse. The maximum ablated depth in the active area from the central pulse (I) is given by *h*_I_. The non-zero ablated depth by pulse (II) in the active area is given by *h*_II_. The zero-ablation depth from pulses outside active (III) area is given by *h*_III_.

The laser ablation model of rectangular shaped cavity including the incubation phenomenon and the saturated ablation depth for multi-pulse processing has been proposed in this work. The graphical representation of a theoretical model in the case of rectangular shaped cavity ablation is given in Fig. 5.

The laser beam is scanned at a certain speed in parallel lines over the copper target, and the rectangular shape cavity is ablated (Fig. 5a). It can be assumed that a single laser pulse ablates the primitive cell marked in a black dashed line (Fig. 5a) with the volume lateral dimensions of Δ*x* and Δ*y* and height of *h*:

6 $${\rm{d}}V=h{\rm{\Delta }}x{\rm{\Delta }}y,$$

**Figure 6:**
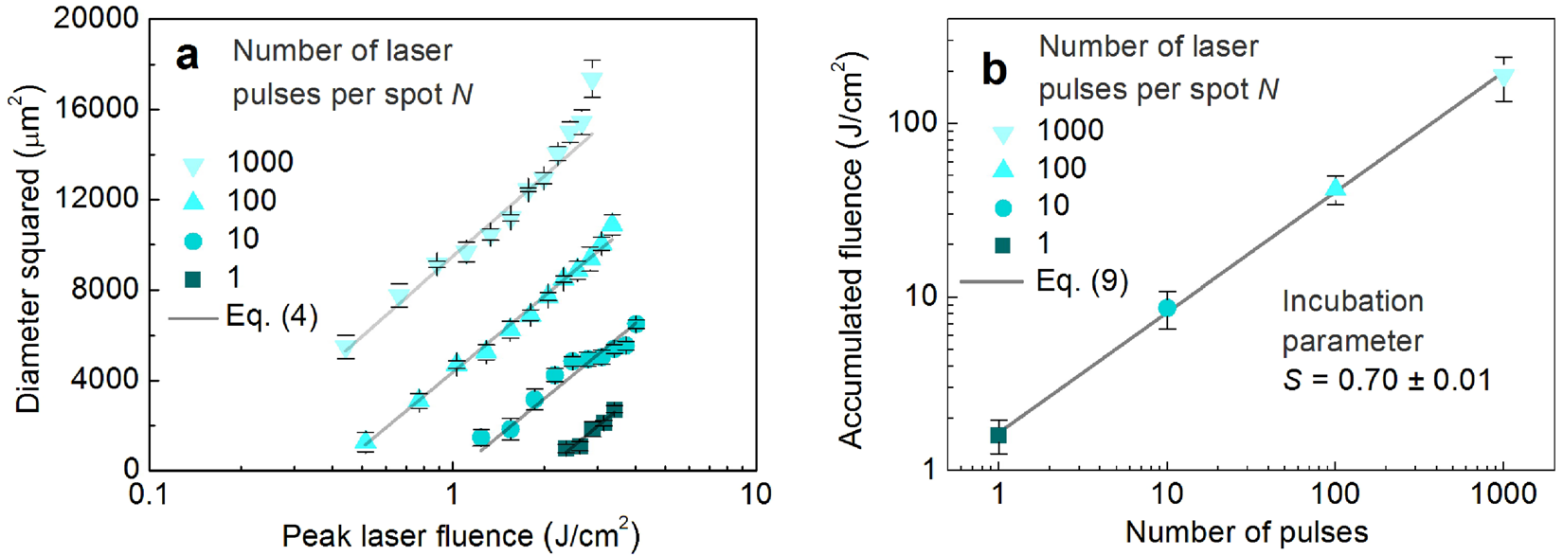
Multi-pulse incubation behaviour of copper after a picosecond laser treatment. (**a**) Squared diameters *D*^2^ of the ablated areas of copper in dependence on the peak laser fluence *F*_0_ at a different number of pulses per spot *N*. Solid dots - experimental data points, solid line - fit by Equation (4). (**b**) Accumulated laser fluence *N* × *F*_th_(*N*) versus a number of laser pulses per spot *N*. Solid dots - experimental data points, solid line - fit by Equation (9). Pulse duration *τ* = 10 ps, laser wavelength *λ* = 1064 nm, repetition rate *f*_p_ = 100 kHz.

The primitive cell is an imaginative concept of the laser ablation volume per single pulse. The volume of the primitive cell is equal to the volume of the rectangular cavity divided by the total number of pulses applied. However, the depth of the primitive cell is influenced by many surrounding pulses within the cut-off radius. The beam scanning path consisting of the parallel scanned lines of the laser beam is shown (Fig. 5b). The three main areas are marked by Roman numerals, and they represent the irradiation points of a different origin: (I) is a selected central point where ablation volume per single pulse is calculated; (II) is any point in the active area pulses have influence in the ablation depth at the (I) position with a non-zero ablation depth; (III) the irradiation points outside the active area laser pulses have zero influence in the ablation depth at the selected point of interest (I). The three-dimensional (3D) representation of a primitive cell of ablated volume per pulse is shown in Fig. 5c. The symbols Δ*h*_I_, Δ*h*_II_ and Δ*h*_III_ represent the ablated depths of three points of interests in Fig. 5b (I), (II) and (III), respectively. The overall ablated depth consists of the ablated depths from pulses from the active area. The total ablation depth per pulse is evaluated by summing all the ablation depths of surrounding irradiated spots in *x* and *y* directions which are in the range of a critical radius *r*_c_:

7 $$h=\sum _{x,y}^{{(x-{x}_{0})}^{2}+{(y-{y}_{0})}^{2}\le {r}_{{\rm{c}}}^{2}}{\rm{\Delta }}{h}_{x,y},$$

the coordinates *x* = Δ*x* · *i* and *y* = Δ*y* · *j*, where *i* and *j* are integer numbers. The coordinates of the transverse pulse positions on the sample are indicated by solid magenta dots (Fig. 5). The ablation rate can be assumed as

8 $$\frac{{\rm{d}}V}{{\rm{d}}t}={f}_{{\rm{p}}}h{\rm{\Delta }}x{\rm{\Delta }}y,$$

where *f*_p_ = 100 kHz is the laser pulse repetition rate. The main purpose of the proposed model is to calculate the ablated volume per pulse of the scanned laser beam by taking into account the incubation phenomenon and saturation of ablation depth for increasing number of pulses per spot. The results of ablation using picosecond laser treatment at different numbers of pulses per spot (*N* = 1, 10, 100, and 1000) are presented in Fig. 6a.

The slope of the straight lines provides information about the Gaussian beam radius *w*_0_ = 41.8 ± 2.1 µm on the target material from Equation (4). With the known beam radius, the peak laser fluence values were calculated by using Equation (3). The parallel lines represent the fixed slopes because of the same spot size on the sample. However, different modification thresholds are observed due to the incubation phenomena. The extrapolation of lines at *D*^2^ = 0 μm gives the ablation threshold fluences: *F*_th_(1) = 2.0 ± 0.35 J/cm^2^; *F*_th_(10) = 0.87 ± 0.21 J/cm^2^; *F*_th_(100) = 0.42 ± 0.08 J/cm^2^; *F*_th_(1000) = 0.19 ± 0.01 J/cm^2^. The copper ablation thresholds correspond well to the multi-pulse ablation thresholds *F*_th_(*N*) = 1.73, 0.74, 0.50, and 0.33 J/cm^2^ for *N* = 1, 10, 100, and 1000 obtained in our previous work (1064 nm, 10 ps, 50 kHz)^27^. The incubation model describes the relation between the single-pulse ablation threshold *F*_th_(1) and the multi-pulse ablation threshold *F*_th_(*N*) in form^39^:

9 $${F}_{{\rm{th}}}(N)={F}_{{\rm{th}}}(1)\cdot {N}^{S-1},$$

with a material-dependent incubation parameter *S* and the laser fluence sufficient to ablate the material for a fixed number of laser pulses per spot *N* is named the ablation threshold fluence *F*_th_(*N*) for a multi-pulse treatment. The accumulated laser fluence *N* × *F*_th_(*N*) versus a number of pulses *N* for copper are given in Fig. 6b. A calculation by Equation (9) yields the accumulation coefficient *S* = 0.70 ± 0.01. The literature values for the accumulation coefficient are: *S* = 0.77 (1064 nm, 10 ps, 50 kHz)^42^; *S* = 0.79 (1064 nm, 10 ps, 50 kHz)^33^; *S* = 0.68 (266 nm, 42 ps, 10 Hz)^59^; *S* = 0.75 (800 nm, 30 fs, 1 kHz)^60^. The accumulation coefficient measured in our work coincides well with the results from other groups. For photochemical as well as for photo-thermal ablation and many materials, a relation of the ablation depth per pulse can be described as^2^:

10 $${\rm{\Delta }}h=\delta \cdot \,\mathrm{ln}(\frac{{F}_{0}}{{F}_{{\rm{th}}}}),$$

where *δ* is the energy penetration depth. If many pulses *N* are applied per single spot, ablation depth *h*(*N*) increases up to a material and laser dependent pulse number *N*_0_ and then reaches saturation^39,43–45,47^. However, an analytical expression is not found in the scientific literature for a prediction of ablation depth growth and saturation with increasing number of pulses per spot. In this work, the analytical function of ablated dimple depth for multi-pulse irradiation has been empirically constructed:

11 $$h(N)=\delta (1)\mathrm{ln}(\frac{{F}_{0}}{{F}_{{\rm{th}}}})[N-{\rm{\Delta }}N\mathrm{ln}(1+{e}^{\frac{N-{N}_{0}}{{\rm{\Delta }}N}})],$$

where *δ*(1) is the energy penetration depth for a single laser pulse, Δ*N* is saturation softness. We have chosen the soft saturation function with linear rise in the initial laser pulses *N* < *N*_0_ − 3Δ*N*, rise and saturation settling in the range *N*_0_ − 3Δ*N* ≤ *N* ≤ *N*_0_ + 3Δ*N*, and saturation after *N* > *N*_0_ + 3Δ*N*. This function is known as softplus function^61^, which becomes a saturation or ramp-saturation function with Δ*N* = 0. The parameters *N*_0_ and *ΔN* have the physical meaning of pulse number required for saturation to begin and pulse number required for saturation settling, respectively. The selected equation (11) has an advantage of having two parameters with defined physical meanings required to describe the saturation phenomenon. Also, it has an analytical form which is advantageous when used in numerical simulations and it is differentiable. The first derivative of softplus is the sigmoid function. The function with initial linear increase has been chosen because straight-line increase in ablation depth versus number of pulses has been experimentally observed in numerous scientific works^62–67^. The saturation after certain number of laser pulses has been also observed experimentally^43,45,46^. Therefore, the empirically selected function (11) fulfilled our needs of a fully analytical expression to be later used for approximation of experimental data, derivation of saturation parameters, and calculation of ablation rates in our numerical modelling.

**Figure 7:**
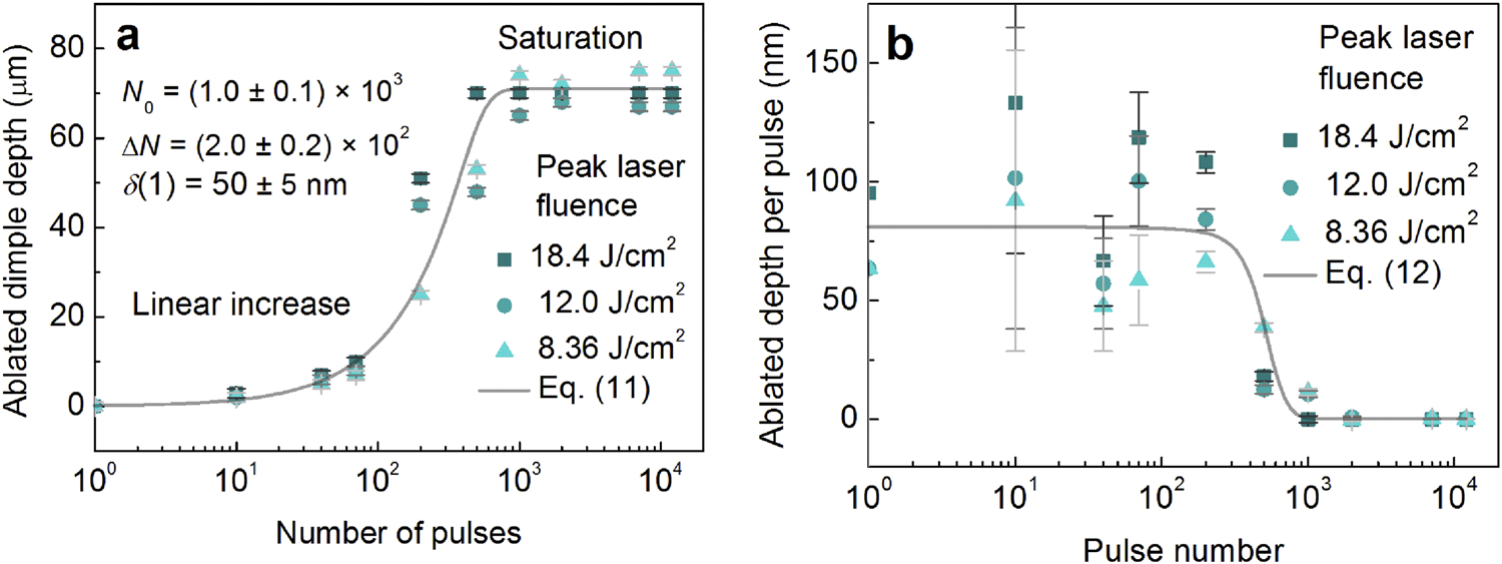
Multi-pulse saturation behaviour of an ablated dimple. (**a**) The ablated depth of dimple dependence on a number of pulses per spot. Solid dots - experimental data points at different peak laser fluence values, solid line - fit by Equation (11) with *N*_0_ = (1.0 ± 0.1) × 10^3^, *ΔN* = (2.0 ± 0.2) × 10^2^ and *δ*(1) ≈ 50 ± 5 nm. (**b**) Ablation depth per pulse dependence on pulse number at different peak laser fluence values. Solid dots - experimental data points, solid line - fit by Equation (12). Pulse duration *τ* = 10 ps, laser wavelength *λ* = 1064 nm, repetition rate *f*_p_ = 100 kHz, peak laser fluences used *F*_0_ = 18.4 J/cm^2^ (*w*_0_ = 21.2 µm), *F*_0_ = 12.0 J/cm^2^ (*w*_0_ = 26.3 µm), and *F*_0_ = 8.36 J/cm^2^ (*w*_0_ = 31.5 µm).

The dimples on a copper target were ablated by using a Gaussian beam by controlling the number of incident pulses per spot. The dimple depth has been experimentally measured by using an optical microscope (Eclipse LV100, Nikon) and the focus knob technique described in^68^. The method is based by the position difference measurement of the top and bottom of the object, using the markings on the fine-focus knob of the microscope. The optical microscope was equipped with *xyz* stage (LV-S64, Nikon) and a 100X microscope objective (LU Plan Fluor 100X, Nikon) with NA = 0.9 and depth of field of 0.19 µm have been used. The *z* accuracy of the fine-focus knob was 1 µm. The depth of field of the objective was much smaller than the accuracy of the fine-focus knob. Therefore, measurement errors of dimple depth were determined by the accuracy of the fine-focus knob. The depth of ablated dimple dependence on the laser pulses per spot is given in Fig. 7a.

The ablated dimple depth increases with the number of pulses applied have been tested using three values of peak laser fluences and similar saturation behaviour has been observed for all fluences (Fig. 7a). The fit of experimental data points by Equation (11) provides: saturation value of *N*_0_ ≈ (1.0 ± 0.1) × 10^3^, the saturation softness of *ΔN* ≈ (2.0 ± 0.2) × 10^2^ pulses, and penetration depth for single pulse irradiation of *δ*(1) ≈ 50 ± 5 nm (Fig. 7a). The fit has a good agreement with the experimental data (Fig. 7a). For small irradiation doses <10^4^ J/cm^2^, the depth of the dimple increases linearly with an increasing number of laser pulses. However, after reaching the saturation value of >10^3^ pulses, the depth of the dimples stops growing and saturates. By differentiating the Equation (11) for a given laser fluence by the number of pulses, the ablated depth per pulse dependence on pulse number is achieved:

12 $${\rm{\Delta }}h(N)=\frac{\partial h(N)}{\partial N}=\frac{\delta (1)}{1+\exp (\frac{N-{N}_{0}}{{\rm{\Delta }}N})}\,\mathrm{ln}(\frac{{F}_{0}}{{F}_{{\rm{th}}}}),$$

The function (12) has constant value for the initial laser pulses *N* < *N*_0_ − 3Δ*N*, followed by the decrease to zero in the range *N*_0_ − 3Δ*N* ≤ *N* ≤ *N*_0_ + 3Δ*N*, and a zero value for the pulse number exceeding *N* > *N*_0_ + 3Δ*N*. This function is known as a sigmoid function^69^ or logistic function and becomes a step or Heaviside function with Δ*N* = 0. The sigmoid functions primitive is the softplus function^61^. The function coincides with scientific works, where constant ablation depth per pulse has been observed experimentally for an initial pulse number^64,70^.

By calculating the first derivative of data points of Fig. 7a, the depth per pulse dependence on the pulse number can be achieved (Fig. 7b). For a large number of laser pulses >10^3^, the ablated depth per pulse has a value close to zero. The data points are scattered from ~50 to ~125 µm. However, its average value stays constant at around Δ*h*(300 < *N*) ≈ 60 µm. The reason for the high scattering of the data points is the large relative errors of the focus-knob method for small depths of <20 µm^71^ (Fig. 7a, *N* < 300) and consequently amplified scattering of its first derivative (Fig. 7b, *N* < 300). However, despite a large scattering of the experimental data points and large measurement errors, the fit of experimental data points by Equation (12) has a good agreement (Fig. 7b).

The penetration depth reduction and saturation behaviour with an increase of number of laser pulses is mostly related to two physical aspects in the keyhole evolution: recast layer formation and multiple reflections. The redeposition of the ablated material and recast layer formation in laser drilling has already been explained in great detail in ref.^72^. Geometrical aspects of deep dimple ablation and multiple reflections influence in keyhole evolution has already been investigated in refs^73,74^ and refs^75–78^, respectively. Therefore, we did not want to go into the physics of already well-known aspects of multi-pulse ablation. Our main goal of this research was to show the responsible combination of physical effects related to the laser ablation and their influence on the material removal rate of the rectangular shape cavity formation. Our approach was to directly compare experimental data with simulation results of the proposed model. However, usage of saturation equation (11) for rectangular cavity ablation with a scanned laser beam, which was derived for dimple ablated with fixed beam position, is not straight forward. Therefore, we provide a graphical scheme and discussion with emphasised similarities between the fixed and scanned beam ablation.

**Figure 8:**
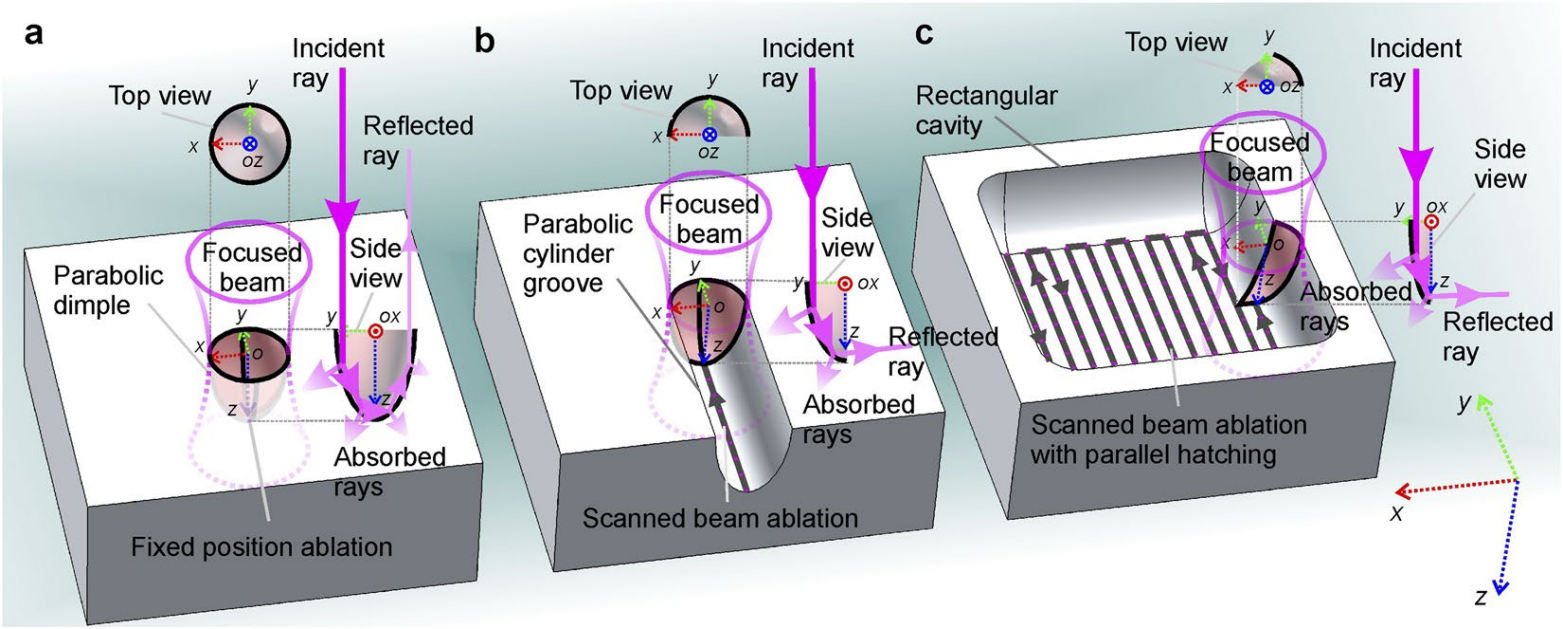
Schematic illustration of the mechanism related to the saturation of the ablation depth in fixed position and beam scanning. (**a**) Dimple with the shape of a paraboloid of revolution ablated by multiple laser pulses with a fixed beam position. (**b**) Groove with the shape of a parabolic cylinder ablated by a scanned laser beam (line scan trajectory). (**c**) Rectangular shaped cavity ablated by a scanned laser beam and parallel line hatching (bidirectional snake scan trajectory). The 3D Cartesian coordinate system *x*, *y*, and *z* denoted by red, green, and blue doted arrows given in (**c**) (right bottom corner) is general for all images (**a**–**c**). The centre positions of the focused Gaussian beam pulses are indicated by letters *o*. The multiple reflections of incident ray from the interior walls are depicted in side views (*yz* planes). The absorption of incident ray and multiply-reflected rays are also depicted in side views (*yz* planes). The increasingly reduced interior wall area interacting with focused Gaussian beams are depicted in top views (*xy* planes) and side views (*yz* planes) as laser-mater interaction area decreases from (**a**) to (**b**) and from (**b**) to (**c**).

The validity conditions of saturation function (11) usage for scanned laser beam which was derived for fixed position ablation is discussed below. There are several known physical aspects related to the saturation behaviour of depth using fixed laser beam position in multi-pulse irradiation, but the most important one is the multiple-reflections of the laser beam from the internal walls of the ablated dimple^46,75,76,79–81^. The Fresnel reflection is drastically increased because of an increase in the incidence angle as shown in Fig. 8a.

The increased reflectivity results in a decrease of absorbed energy. Also, an internal surface area of the dimple is larger than an irradiated disk on a flat surface in the initial stage of ablation. The increased beam-matter interaction surface area causes the decreased energy density on the internal surface walls of a dimple (Fig. 8a). As the result, the dimple depth stops increasing for the following laser pulses and the saturation starts. With the fixed laser beam position, the beam interacts with interior walls of the parabolic dimple (Fig. 8a). If compared to the scanned laser beam and grove ablation with the shape of the parabolic cylinder depicted in Fig. 8b, the direct beam interaction area is about a half of the internal paraboloid area shown in Fig. 8a. In the rectangular shaped cavity formation by bidirectional scanning and hatching the beam interaction area is even smaller as illustrated in Fig. 8c. However, the same effect of increase in incidence angle, its related increase of Fresnel reflection, and decrease of absorption are observed in all three situations. The effect of decrease of energy density on the ablated surface is common for the fixed and scanned beam ablation. Only the magnitude of the effect is different, which is related to the geometrical aspects of ablated cavities, for all three cases. The similarities presented in Fig. 8 justifies that equation (11) derived for a fixed position is valid for a scanned laser beam, with some tolerance. Therefore, having in mind that fixed position and scanned beam ablation are different procedures, only having similarities in saturation behaviour, the formula (11) will be used for numerical simulation in our model.

The model incorporated a numerical calculation of an equation system with the input of the copper ablation parameters. All laser ablation parameters required for the proposed ablation model of the rectangular shape cavity are given in Table 1.

**Table 1:** Irradiation parameters used for numerical simulation of the ablation model.

Parameter	Symbol	Value	Unit
Saturation value	*N*_0_	(1.0 ± 0.1) × 10^3^	pulses
Saturation softness	Δ*N*	(2.0 ± 0.2) × 10^2^	pulses
Incubation parameter	*S*	0.70 ± 0.01	a.u.
Ablation threshold for single pulse irradiation	*F*_th_(1)	2.0 ± 0.35	J/cm^2^
Penetration depth for single pulse irradiation	*δ*(1)	50 ± 5	nm

For the selected point of interest (I) (Fig. 5b) the active circle area $\pi {r}_{{\rm{c}}}^{2}$ with the cut-off radius *r*_c_. All the laser pulses within this radius have non-zero ablation depths in position (I) (Fig. 5b). Ablation will not be possible by the laser pulses, which centre positions *x* and *y* are outside of cut-off radius as (*x* − *x*_0_)^2^ + (*y* − *y*_0_)^2^ > *r*_c_^2^, because laser fluence from the Gaussian wings will be below the threshold for multi-pulse ablation. The number of all those pulses *N* within the cut-off radius can be evaluated as a ratio between the active circle area and the primitive cell base area:

13 $$N\approx \frac{\pi {r}_{{\rm{c}}}^{2}}{{\rm{\Delta }}x{\rm{\Delta }}y},$$

where Δ*x* and Δ*y* are the distance of the irradiation spots in the transverse directions *x* and *y*, respectively. On the other hand, the cut-off *r*_c_ of the ablated area evaluated by taking into account that in the processed area *N* pulses are absorbed, and the ablation threshold is decreased. From Equations (4) and (9):

14 $${r}_{{\rm{c}}}={w}_{0}\sqrt{\frac{1}{2}\,\mathrm{ln}(\frac{{F}_{0}}{{F}_{{\rm{th}}}(1)\cdot {N}^{S-1}})},$$

By numerically solving Equations (13) and (14) the cut-off radius and the number of laser pulses per spot can be evaluated. The inter-pulse distance Δ*x* and the distance between scanned lines Δ*y* have been chosen with equal values in order to reduce the number of laser processing parameters. Moreover, they can be replaced by an experimentally controllable processing parameter – a beam scanning speed *v* on the sample:

15 $${\rm{\Delta }}x={\rm{\Delta }}y=\frac{v}{{f}_{{\rm{p}}}},$$

where *f*_p_ is the pulse repetition rate. Therefore, the main well known equations, defining the laser ablation by a Gaussian laser beam were included into this model: diameter squared of the ablated crater dependence on the peak laser fluence by Equation (4); incubation behaviour for multi-pulse treatment by Equation (9); ablation depth dependence on the laser fluence by Equation (10); saturation of ablation depth by Equation (11).

## Results

### Experimental and modelling results of rectangular cavity ablation

**Figure 9:**
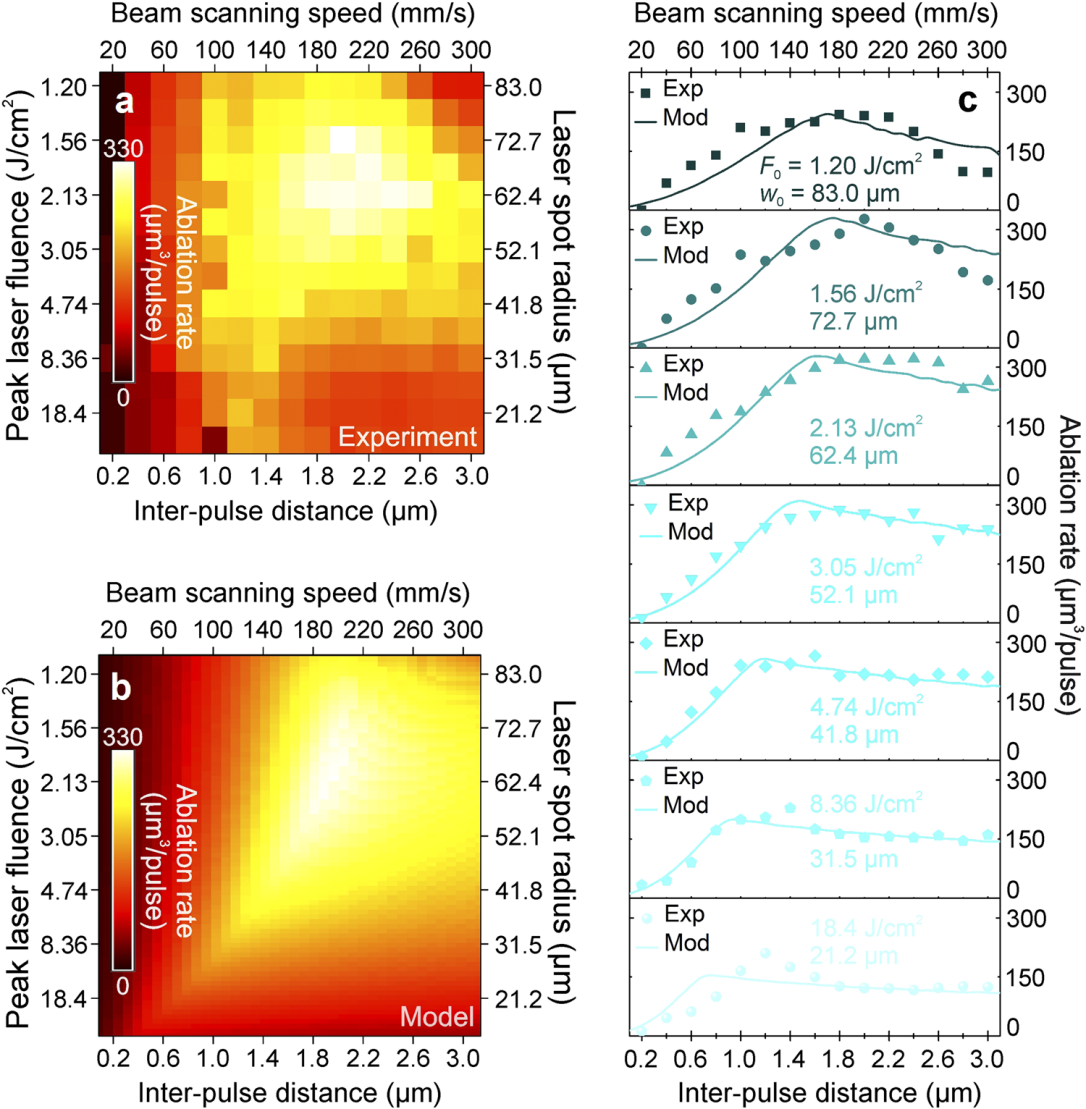
Comparison of the experimental data to the theoretical model. Laser ablation rate (colour scale) of the copper dependence on the peak laser fluence (left axis), laser spot radius (right axis), inter-pulse distance (bottom axis), and the beam scanning speed (top axis): (**a**) data from the experiment; (**b**) results from the new rectangular shaped cavity ablation model; (**c**) the ablation rate dependence on the inter-pulse distance (bottom axis) and the beam scanning speed (top axis) at different peak laser fluences ranging from 1.20 J/cm^2^ (top) to 18.4 J/cm^2^ (bottom) and laser spot radiuses ranging from 21.2 μm (bottom) to 83.0 μm (top). Experimental data points (solid dots) are quantitatively compared with the predictions of our theoretical model (solid lines). Laser parameters: pulse duration 10 ps; wavelength 1064 nm; pulse energy 130 μJ; pulse repetition rate 100 kHz; average laser power 13.0 W.

The ablation rate has been tested experimentally and theoretically at various beam scanning speeds (inter-pulse delays) and spot radiuses on the sample (peak laser fluences) (for details see Materials and Methods). The ultrafast laser with pulse duration of 10 ps, irradiation wavelength of 1064 nm, pulse energy of 130 μJ, and pulse repetition rate of 100 kHz was used to ablate an array of rectangular areas in the copper plate (for details see Materials and Methods), and ablation rate was measured (Fig. 9a).

Equations (7–9 and 12–15) have been solved numerically by using the measured ablation characteristics of copper from Table 1 and symbolic/numeric computing software (Maple, Maplesoft). The calculated ablation rate versus laser processing parameters is given in (Fig. 9b). The experimental data of measured ablation rates was quantitatively compared to the numerical results of the model equations in Fig. 9c. The series of cross sections from the experiment and model with variable peak laser fluence (from 1.20 J/cm^2^ to 18.4 J/cm^2^) and laser spot radius (from 21.2 μ to 83.0 μm) are shown as a function of inter-pulse distance. Our simulation results at each value of the laser fluence were in good agreement with the ablation rate measurements. The experimental data points can be well described by the modelling curves that take into account the incubation effect and saturation of ablation depth for multi-pulse irradiation. At a low scan speed *v* < 100 mm/s, a high number of pulses per spot is achieved because of the high pulse overlap. Therefore, the ablation threshold decreases drastically, and its related total ablated depth increases. However, the saturation of ablation depth is achieved, and the ablated depth is not increased any more. The ablation rate reaches moderate values and decreases even more by reducing the scanning speed. Meanwhile, with high scanning speed *v* > 280 mm/s, the number of laser pulses per spot becomes small because of the low pulse overlap. Therefore, the ablation threshold increases due to the accumulation effect and it causes a small ablation depth. Contrary, the large ablated transverse area for a single pulse is caused by a large inter-pulse distance. The product of a small total ablation depth and large transverse area gives only moderate ablation rate.

For small laser spot sizes *w*_0_ < 40 μm, the laser fluence high above the ablation threshold is achieved, which should result in large ablation depths. However, low numbers of pulses per spot are achieved even for small inter-pulse distances. Therefore, low ablation rates are achieved because of high ablation thresholds. For high laser spot sizes *w*_0_ > 75 μm, it is difficult to reach fluence above the ablation threshold, so low ablation depths are achieved, as well. However, the number of laser pulses is sufficiently high even for large scanning speed because of the high pulse overlap. Thus, low and moderate ablation rates are achieved.

The optimal laser parameter set exists for maximum available ablation rate: scanning speed 180 mm/s < *v* < 220 mm/s and beam spot radius 60 μm < *w*_0_ < 70 μm (Fig. 9). In the optimal set up the maximal ablated volume per pulse is reached because of a combination of all the factors influencing material removal rate. The moderate scanning speeds and its related distance between adjacent pulses give sufficiently large transverse ablation area for a single pulse. Also, the moderated spot size gives the sufficiently high number of laser pulses and its related appreciable decrease of ablation threshold due to the accumulation effects. Volume pulses per unit point are less, so the result is a high ablation threshold, which decreases increasing the number of pulses. The saturation depth is just reached in this processing region. Therefore, the ablation rate has a maximum at scanning speed of *v*_max_ ≈ 200 mm/s and a laser spot radius of *w*_0 max_ ≈ 62.4 μm (Fig. 9). The experiment data results have a good agreement with the numerical calculation results of the new model.

By having laser pulse energy *E*_p_ = 130 μJ and spot radius *w*_0_ = 62.4 μm the calculated peak laser fluence Equation (3) with laser pulse energy is *F*_0_ = 2.13 J/cm^2^. By solving Equations (13) and (14) using optimal inter-pulse distances between scanned spots and scanned lines of Δ*x* = Δ*y* = 2.0 μm, one can calculate the cut-off radius *r*_c_ ≈ 62.7 μm and a number of pulses per spot *N* ≈ 2.5 × 10^3^ pulses. The threshold for that number of pulses by Equation (9) and accumulation coefficient of *S* = 0.70 is *F*_th_(2.5 × 10^3^) = 0.28 J/cm^2^. The ratio between the optimal laser fluence and the threshold is 7.6. This number is close to *e*^2^ ≈ 7.4 which is theoretically predicted^26,27^.

The experimental measurement of ablation rate was based on the rectangular shape cavity profiles that were achieved by a stylus profiler and average depth evaluation. The measurement errors were related to the bottom surface roughness of an ablated cavity. The average relative error of a cavity depth and its related ablation rate measurement was of 2.5%. The peak laser fluence can be calculated by Equation (3) and is proportional to the laser power and inversely proportional to the spot radius squared. The laser power was measured by a power measurement unit (for details see Laser beam characterization, Materials and Methods) with a relative error of 0.7%. The beam radiuses on the sample at each height of the specimen were measured by a technique described in^51^ (Fig. 3). The average relative error of the beam radius measurement was 2.7%. The scanning speed (inter-pulse distance) and its related pulse overlap were controlled by a galvanometer scanner with an accuracy of 1.0%^82,83^. The ablation rate model with a separate variation of laser fluence, beam radius and scanning speed (inter-pulse distance) by 0.7%, 2.7% and 1.0% gave the highest relative errors of 0.4%, 1.5% and 0.8%, respectively. The ablation model errors were always comparable with the experimental errors. The largest relative errors of the model calculations of ablation rate of 1.5% were achieved when spot radius was varied. The spot size variation influenced the calculations mostly because it has the direct influence on several physical parameters: peak laser fluence by Equation (3) and cut-off radius by Equation (14). Moreover, peak laser fluence is directly involved in the ablation depth per pulse calculations by Equation (12). The largest relative variation of the calculated ablation rate of 1.5% is smaller than the experimental error of 2.5%.

### Efficient deep laser engraving and structuring

**Figure 10:**
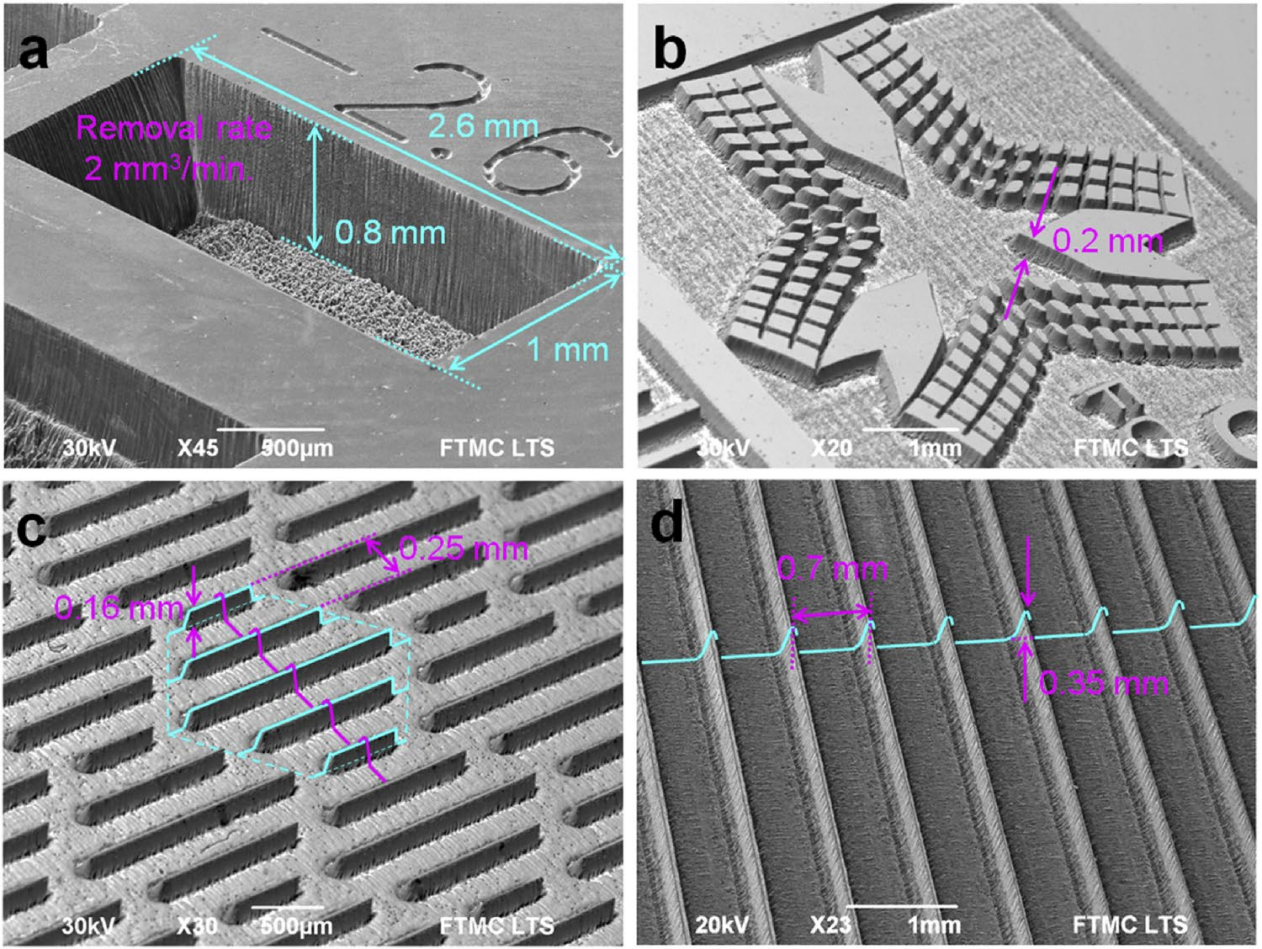
Examples of efficient surface structuring by laser. SEM images of structures ablated in copper by using a picosecond laser: (**a**) rectangular cavity with dimensions 2.6 mm × 1.0 mm × 0.8 mm processed in 1 min time; (**b**) logo of Center for Physical Sciences and Technology, engraving depth 0.2 mm; (**c**) bio-inspired shark skin-like structure, period 0.25 mm, blade height 0.16 mm; (**d**) drag reducing blade-riblet structure: period 0.7 mm; blade height 0.35 mm. Laser processing parameters: wavelength of irradiation 1064 nm; mean laser power 13.0 W; pulse repetition rate 100 kHz; pulse duration 10 ps; scanning speed 200 mm/s; spot radius *w*_0_ = 62.4 μm, peak laser fluence *F*_0_ = 2.13 J/cm^2^, copper removal rate of 2.0 mm^3^/min.

One of the aims of the study was to show that the laser ablation is capable of 2.5D structure formation and replication of bio-inspired structure at high speed. The copper was structured by using pulsed laser irradiation with picosecond laser duration (for details see Experimental setup and procedures, Materials and Methods). The examples of deep laser engraving and 2.5D structuring are given in Fig. 10.

The laser ablated cavity with the removal rate of 330 μm^3^/pulse has been performed by using 130 μJ laser pulse energy Fig. 10a. The current world record of ablation cooled process by bursts of laser pulses is ~600 μm^3^/pulse for 130 μJ total incident energy of 200 pulses in the burst at 864 MHz^3^. The achieved removal rate of efficient ablation in our experiments for copper is ~2 times slower than the record obtained with a specially designed GHz burst laser^3^. The efficient ablation laser parameter set has been used to create a rectangular cavity (Fig. 10a), the logo of Center for Physical Sciences and Technology (Fig. 10b), a bio-inspired shark skin structure (Fig. 10c) and a drag reducing blade-riblet structure (Fig. 10d) in the copper sample. The ablation speed of 2.0 mm^3^/min has been achieved at an average laser power of 13.0 W.

## Discussion and Conclusions

Scientific background of this research was two previous works^26,27^ that emerged a decade ago attempting to predict efficient laser ablation. However, the accumulation effect and saturation of ablation depth for multi-pulse ablation was not taken into account. In this research, the complete model of laser ablation incorporating the accumulation effect and the saturation of ablation depth, if many pulses are applied to a single spot, is presented. Laser irradiation with picosecond pulse duration has been used for the ablation of the target material of copper. The processing parameters: scanning speed (inter-pulse distance, distance between scanned lines) and spot size on the sample (peak laser fluence) has been varied in order to reach maximum removal rate. The new proposed model of rectangular shape cavity ablation has been created which takes into account the ablation threshold and the penetration depth decrease with increasing number of laser pulses per spot. The model also assumes that laser beam has a Gaussian intensity profile, the ablation depth per pulse is proportional to the logarithm of fluence applied with a certain ablation threshold, the beam is scanned on a flat target material surface by parallel overlapping lines, the inter-pulse distance is equal to the distance between scanned lines, and the ablation volume per pulse is calculated by summing ablated depths from surrounding pulses within cut-off radius. The experiments have been conducted for the ablation rate measurements and confirmation of modelling results. The experimental evidence of the model is clearly seen in Fig. 9 as the numerical simulation of the model equations is in good agreement with experimental results. Thus, the modelling results are confirmed by the experimental data. The maximum removal rate of 2.0 mm^3^/min (330 μm^3^/pulse) has been achieved for the copper target by using the laser processing parameters: wavelength of irradiation 1064 nm; mean laser power 13.0 W; pulse repetition rate 100 kHz; pulse duration 10 ps; inter-pulse distance 2.0 µm (scanning speed 200 mm/s); peak laser fluence 2.13 J/cm^2^ (spot radius *w*_0_ = 62.4 μm). From a scientific point of view, it is worth mentioning that the incorporation of the accumulation effect and saturation of ablation depth for multi-pulse ablation to laser ablation model has been demonstrated for the first time. Comparing our model to previous attempts of predicting the efficient ablation point, where only logarithm dependence on the fluence of ablation depth and diameter squared for ablation by Gaussian beam has been included, our model is much more complete. From an engineering point of view, it is essential to have a theoretical tool and laser scanning procedure to find the most efficient ablation spot. In this work, theoretical model and laser scanning procedure are presented enabling high texturing speed. The possibility to mimic the bio-inspired functional surfaces at high processing rate has been demonstrated. The results of laser application contribute to technological conclusions, that controlled laser irradiation is a reliable tool to mimic functional surfaces found in nature.

## References

[1] Preuss S, Demchuk A, Stuke M (1995). Sub-picosecond UV laser ablation of metals. Appl. Phys. A Mater. Sci. Process..

[2] Chichkov BN, Momma C, Nolte S, von Alvensleben F, Tünnermann A (1996). Femtosecond, picosecond and nanosecond laser ablation of solids. Appl. Phys. A Mater. Sci. Process..

[3] Kerse C (2016). Ablation-cooled material removal with ultrafast bursts of pulses. Nature.

[4] Tang M, Shim V, Pan ZY, Choo YS, Hong MH (2011). Laser ablation of metal substrates for super-hydrophobic effect. J. Laser Micro Nanoen..

[5] Luo F (2014). Enhancement of pulsed laser ablation in environmentally friendly liquid. Opt. Express.

[6] Her TH, Finlay RJ, Wu C, Deliwala S, Mazur E (1998). Microstructuring of silicon with femtosecond laser pulses. Appl. Phys. Lett..

[7] Zhu S, Lu YF, Hong MH (2001). Laser ablation of solid substrates in a water-confined environment. Appl. Phys. Lett..

[8] Takahashi F (2016). Picosecond optical vortex pulse illumination forms a monocrystalline silicon needle. Sci. Rep..

[9] Gattass RR, Mazur E (2008). Femtosecond laser micromachining in transparent materials. Nat. Photonics.

[10] Wang ZB (2003). Femtosecond laser ablation of polytetrafluoroethylene (Teflon) in ambient air. J. Appl. Phys..

[11] Mirza I (2016). Ultrashort pulse laser ablation of dielectrics: Thresholds, mechanisms, role of breakdown. Sci. Rep..

[12] Phillips KC, Gandhi HH, Mazur E, Sundaram SK (2015). Ultrafast laser processing of materials: a review. Adv. Opt. Photonics.

[13] Bityurin N, Luk’yanchuk BS, Hong MH, Chong TC (2003). Models for laser ablation of polymers. Chem. Rev..

[14] Malinauskas M (2016). Ultrafast laser processing of materials: from science to industry. Light Sci. Appl..

[15] Chong TC, Hong MH, Shi LP (2010). Laser precision engineering: From microfabrication to nanoprocessing. Laser Photonics Rev..

[16] Plech A, Kotaidis V, Lorenc M, Boneberg J (2006). Femtosecond laser near-field ablation from gold nanoparticles. Nat. Phys..

[17] Ye S, Cao Q, Wang Q, Wang T, Peng Q (2016). A highly efficient, stable, durable, and recyclable filter fabricated by femtosecond laser drilling of a titanium foil for oil-water separation. Sci. Rep..

[18] Öktem B (2013). Nonlinear laser lithography for indefinitely large-area nanostructuring with femtosecond pulses. Nat. Photonics.

[19] Du Z, Chen L, Kao TS, Wu M, Hong M (2015). Improved optical limiting performance of laser-ablation-generated metal nanoparticles due to silica-microsphere-induced local field enhancement. Beilstein J. Nanotechnol..

[20] Zhu S, Lu YF, Hong MH, Chen XY (2001). Laser ablation of solid substrates in water and ambient air. J. Appl. Phys..

[21] Chen GX (2004). Preparation of carbon nanoparticles with strong optical limiting properties by laser ablation in water. J. Appl. Phys..

[22] Berns MW (1981). Laser Microsurgery in Cell and Developmental Biology. Science..

[23] Graydon O (2015). Laser ablation: Unblocking arteries. Nat. Photonics.

[24] Chen H, Li H, Sun Y, Wang Y, Lü P (2016). Femtosecond laser for cavity preparation in enamel and dentin: ablation efficiency related factors. Sci. Rep..

[25] Yuan F (2016). An automatic tooth preparation technique: A preliminary study. Sci. Rep..

[26] Furmanski J, Rubenchik AM, Shirk MD, Stuart BC (2007). Deterministic processing of alumina with ultrashort laser pulses. J. Appl. Phys..

[27] Račiukaitis G, Brikas M, Gečys P, Voisiat B, Gedvilas M (2009). Use of High Repetition Rate and High Power Lasers in Microfabrication: How to Keep the Efficiency High?. J. Laser Micro Nanoen..

[28] Lauer B, Jaeggi B, Neuenschwander B (2014). Influence of the pulse duration onto the material removal rate and machining quality for different types of steel. Phys. Procedia.

[29] Neuenschwander B (2016). Laser surface structuring with 100 W of average power and sub-ps pulses. J. Laser Appl..

[30] Neuenschwander B, Jaeggi B, Zimmermann M, Penning L, DeLoor R (2014). High Throughput Laser Processing with Ultra-Short Pulses by High Speed Line-Scanning in Synchronized Mode. Proc. CLEO.

[31] Neuenschwander B, Kramer T, Lauer B, Jaeggi B (2015). Burst mode with ps- and fs-pulses: Influence on the removal rate, surface quality, and heat accumulation. Proc. SPIE.

[32] Neuenschwander B, Jaeggi B, Schmid M, Hennig G (2014). Surface structuring with ultra-short laser pulses: Basics, limitations and needs for high throughput. Phys. Procedia.

[33] Jaeggi B (2011). Influence of the pulse duration in the ps-regime on the ablation efficiency of metals. Phys. Procedia.

[34] Neuenschwander B (2010). Processing of metals and dielectric materials with ps-laserpulses: results, strategies, limitations and needs. Proc. SPIE.

[35] Neuenschwander B (2013). Factors controlling the incubation in the application of ps laser pulses on copper and iron surfaces. Proc. SPIE.

[36] Du K (2009). Thin layer ablation with lasers of different beam profiles: energy efficiency and over filling factor. Proc. SPIE.

[37] Kramer T (2016). Influence of Pulse Bursts on the Specific Removal Rate for Ultra-fast Pulsed Laser Micromachining of Copper. Phys. Procedia.

[38] Bass M, Barrett HH (1973). Laser-induced Damage Probability at 1.06 microm and 0.69 microm. Appl. Opt..

[39] Jee Y, Becker FM, Walser MR (1988). Laser-induced damage on single-crystal metal surfaces. J. Opt. Soc. Am. B.

[40] Bobrinetskiy I (2017). Thermal and fluid processes of a thin melt zone An abnormal non-incubation effect in femtosecond laser processing of freestanding reduced graphene oxide paper. J. Phys. D Appl. Phys..

[41] Di Niso F (2014). Role of heat accumulation on the incubation effect in multi-shot laser ablation of stainless steel at high repetition rates. Opt. Express.

[42] Raciukaitis G, Brikas M, Gecys P, Gedvilas M (2008). Accumulation effects in laser ablation of metals with high-repetition- rate lasers. Proc. SPIE.

[43] Lukac N, Suhovršnik T, Lukac M, Jezeršek M (2016). Ablation characteristics of quantum square pulse mode dental erbium laser. J. Biomed. Opt..

[44] Cangueiro LT, Vilar R, Botelho do Rego AM, Muralha VSF (2012). Femtosecond laser ablation of bovine cortical bone. J. Biomed. Opt..

[45] Ji L (2012). Ti: Sapphire femtosecond laser ablation of dental enamel, dentine, and cementum. Lasers Med. Sci..

[46] Li F (2017). Technology Nanosecond laser ablation of Al-Si coating on boron steel. Surf. Coat. Technol..

[47] Domke M (2015). Controlling depth and distance of the hole formations at the bottom of laser-scribed trenches in silicon using fs-pulses. Proc. SPIE.

[48] Suzaki Y, Tachibana A (1975). Measurement of the μm sized radius of Gaussian laser beam using the scanning knife-edge. Appl. Opt..

[49] Veshapidze G, Trachy ML, Shah MH, DePaola BD (2006). Reducing the uncertainty in laser beam size measurement with a scanning edge method. Appl. Opt..

[50] Díaz-Uribe R, Rosete-Aguilar M, Ortega-Martinez R (1993). Position sensing of a Gaussian beam with a power meter and a knife edge. Rev. Mex. Fis.

[51] Liu MJ (1982). Simple technique for measurements of pulsed Gaussian-beam spot sizes. Opt. Lett..

[52] Spellauge M (2018). Ultra-short-pulse laser ablation and modification of fully sprayed single walled carbon nanotube networks. Carbon..

[53] Dick- PK, Hildenha- DJ (2011). Micromachining using high-power picosecond lasers Comparison of various materials. Micro Mater. Process..

[54] Farson DF (2008). Femtosecond laser micromachining of dielectric materials for biomedical applications. J. Micromechanics Microengineering.

[55] Cirp P (2018). Influence of laser spot size and shape on ablation efficiency using ultrashort pulse laser system. Procedia CIRP.

[56] Phipps CR, Llc PA, Luke J (2004). Micropropulsion Using a Laser Ablation Jet. J. Propul. Power.

[57] Lednev VN, Pershin SM, Obraztsova ED, Kudryashov SI, Bunkin AF (2013). Single-shot and single-spot measurement of laser ablation threshold for carbon nanotubes. J. Phys. D. Appl. Phys..

[58] Sun H (1998). Thin lens equation for a real laser beam with weak lens aperture truncation. Opt. Eng..

[59] Huynh TTD, Semmar N (2014). Dependence of ablation threshold and LIPSS formation on copper thin films by accumulative UV picosecond laser shots. Appl. Phys. A Mater. Sci. Process..

[60] Nathala CSR (2016). Ultrashort laser pulse ablation of copper, silicon and gelatin: effect of the pulse duration on the ablation thresholds and the incubation coefficients. Appl. Phys. A Mater. Sci. Process..

[61] Dugas, C., Bengio, Y., Belisle, F., Nadeau, C. & Garcia, R. Incorporating Second-Order Functional Knowledge for Better Option Pricing. *Proc. NIPS* 472–478 (2001).

[62] Kautek, W. & Armbruster, O. In *Lasers in Materials Science*, Springer Series *in Materials Science* (eds Castillejo, M., Ossi, P. M. & Zhigilei, L.) 43–66 (Springer International Publishing, 2014).

[63] Kautek W (1996). Laser ablation of dielectrics with pulse durations between 20 fs and 3 ps. Appl. Phys. Lett..

[64] Delmdahl R, Paetzel R (2014). Laser Drilling of High-Density Through Glass Vias (TGVs) for 2.5D and 3D Packaging. J. Microelectron. Packag. Soc..

[65] Daengngam C (2015). Fabrication and characterization of periodically patterned silica fiber structures for enhanced second-order nonlinearity. Opt. Express.

[66] Dorronsoro C, Siegel J, Remon L, Marcos S (2008). Suitability of Filofocon A and PMMA for experimental models in excimer laser ablation refractive surgery. Opt. Express.

[67] Ashforth SA, Simpson MC, Bodley O, Oosterbeek R (2015). Ultrashort pulse laser interactions with cortical bone tissue for applications in orthopaedic surgery. Proc. SPIE.

[68] Brattgård S (1954). Microscopical determinations of the thickness of histological sections. J. R. Microsc. Soc..

[69] Han J, Moraga C (1995). The Influence of the Sigmoid Function Parameters on the Speed of Backpropagation Learning. Proc. IWANN.

[70] Schille J, Ebert R, Loeschner U, Scully P (2010). High repetition rate femto second laser processing of metals. Proc. SPIE.

[71] Harris RM (1985). Light microscopic depth measurements of thick sections. J. Neurosci. Methods.

[72] Schulz, W. & Eppelt, U. In *The Theory of Laser Materials Processing: Heat and Mass Transfer in Modern Technology* (eds. Dowden, J. & Schulz, W.) 129–166 (Springer International Publishing, 2009).

[73] Yung KC, Zhu HH, Yue TM (2005). Theoretical and experimental study on the kerf profile of the laser micro-cutting NiTi shape memory alloy using 355 nm Nd:YAG. Smart Mater. Struct..

[74] Pocorni J, Petring D, Powell J, Deichsel E, Kaplan AFH (2015). The Effect of Laser Type and Power on the Efficiency of Industrial Cutting of Mild and Stainless Steels. J. Manuf. Sci. Eng..

[75] Ki H, Mohanty PS, Mazumder J (2002). Multiple reflection and its influence on keyhole evolution. J. Laser Appl..

[76] Cho JH, Na SJ (2006). Implementation of real-time multiple reflection and Fresnel absorption of laser beam in keyhole. J. Phys. D. Appl. Phys..

[77] Courtois M, Carin M, Le Masson P, Gaied S, Balabane M (2014). A complete model of keyhole and melt pool dynamics to analyze instabilities and collapse during laser welding. J. Laser Appl..

[78] Döring S, Richter S, Nolte S, Tünnermann A (2010). *In situ* imaging of hole shape evolution in ultrashort pulse laser drilling. Opt. Express.

[79] Wu D, Hua X, Li F, Huang L (2017). Understanding of spatter formation in fiber laser welding of 5083 aluminum alloy. Int. J. Heat Mass Transf..

[80] Courtois M, Carin M, Masson P, Le, Gaied S (2013). A new approach to compute multi-reflections of laser beam in a keyhole for heat transfer and fluid flow modelling in laser welding. J. Phys. D Appl. Phys..

[81] Pang S, Chen W, Zhou J, Liao D (2014). Self-consistent modeling of keyhole and weld pool dynamics in tandem dual beam laser welding of aluminum alloy. J. Mater. Process. Tech..

[82] Bechtold P, Hohenstein R, Schmidt M (2013). Evaluation of disparate laser beam deflection technologies by means of number and rate of resolvable spots. Opt. Lett..

[83] Römer GRBE, Bechtold P (2014). Electro-optic and acousto-optic laser beam scanners. Phys. Procedia.

